# Age groups that sustain resurging COVID-19 epidemics in the United States

**DOI:** 10.1126/science.abe8372

**Published:** 2021-02-02

**Authors:** Mélodie Monod, Alexandra Blenkinsop, Xiaoyue Xi, Daniel Hebert, Sivan Bershan, Simon Tietze, Marc Baguelin, Valerie C. Bradley, Yu Chen, Helen Coupland, Sarah Filippi, Jonathan Ish-Horowicz, Martin McManus, Thomas Mellan, Axel Gandy, Michael Hutchinson, H. Juliette T Unwin, Sabine L. van Elsland, Michaela A. C. Vollmer, Sebastian Weber, Harrison Zhu, Anne Bezancon, Neil M. Ferguson, Swapnil Mishra, Seth Flaxman, Samir Bhatt, Oliver Ratmann, on behalf of the Imperial College COVID-19 Response Team

**Affiliations:** 1 Department of Mathematics, Imperial College London, London, UK.; 2 Foursquare Inc, New York, NY, USA.; 3 Emodo, San Francisco, CA, USA.; 4 MRC Centre for Global Infectious Disease Analysis; and the Abdul Latif Jameel Institute for Disease and Emergency Analytics (J-IDEA), School of Public Health, Imperial College London, London, UK.; 5 Novartis Pharma AG, Basel, Switzerland.; 6 Department of Statistics, University of Oxford, Oxford, UK.; 7 Section of Epidemiology, Department of Public Health, University of Copenhagen, Denmark.

## Abstract

Following initial declines, in mid 2020 a resurgence in transmission of novel coronavirus disease (COVID-19) occurred in the US and Europe. As COVID19 disease control efforts are re-intensified, understanding the age demographics driving transmission and how these affect the loosening of interventions is crucial. We analyze aggregated, age-specific mobility trends from more than 10 million individuals in the US and link these mechanistically to age-specific COVID-19 mortality data. We estimate that as of October 2020, individuals aged 20-49 are the only age groups sustaining resurgent SARS-CoV-2 transmission with reproduction numbers well above one, and that at least 65 of 100 COVID-19 infections originate from individuals aged 20-49 in the US. Targeting interventions – including transmission-blocking vaccines – to adults aged 20-49 is an important consideration in halting resurgent epidemics and preventing COVID-19-attributable deaths.

Following worldwide spread of the novel severe acute respiratory syndrome coronavirus 2 (SARS-CoV-2), the implementation of large-scale non-pharmaceutical interventions has led to sustained declines in the number of reported SARS-CoV-2 infections and deaths from coronavirus disease 2019 (COVID-19) (*1*, *2*). However, since mid-June 2020, the daily number of reported COVID-19 cases is resurging in Europe and North America, and surpassed in the United States alone 40,000 daily reported cases on June 26, and 100,000 on November 4 2020 (*3*). Demographic analyses have shown that the share of individuals aged 20-29 among reported cases increased most, suggesting that young adults may be driving re-surging epidemics (*4*). However reported COVID-19 case data may not be a reliable indicator of disease spread due to the large proportion of asymptomatic COVID-19, increased testing, and changing testing behavior (*5*). Here, we use detailed, longitudinal, and age-specific population mobility and COVID-19 mortality data to estimate how non-pharmaceutical interventions, changing contact intensities, age, and other factors have interplayed and led to resurgent disease spread. We test previous claims that resurgent COVID-19 is a result of increased spread from young adults, identify the population age groups driving SARS-CoV-2 spread across the US through October 29, 2020, and quantify changes in transmission dynamics since schools reopened.

Similar to many other respiratory diseases, the spread of SARS-CoV-2 occurs primarily through close human contact, which, at a population level, is highly structured (*6*). Prior to the implementation of COVID-19 interventions, contacts concentrated among individuals of similar age, were highest among school-aged children and teens, and also common between children/teens and their parents, and middle-aged adults and the elderly (*6*). Since the beginning of the pandemic, these contact patterns have changed substantially (*7*–*9*). In the US, the Berkeley Interpersonal Contact Study indicates that in late March 2020 after stay-at-home orders were issued, the average number of daily contacts made by a single individual, also known as contact intensity, dropped to four or fewer contacts per day (*9*). Data from China show that infants and school-aged children and teens had almost no contact to similarly aged children and teens in the first weeks after stay-at-home orders, and reduced contact intensities with older individuals (*7*). However, detailed human contact and mobility data have remained scarce, especially longitudinally, although such data are essential to better understand the engines of COVID-19 transmission (*10*).

## Cell-phone data suggest similar rebounds in mobility across age groups

We compiled a national-level, aggregate mobility data set using cell phone data from >10 million individuals with Foursquare’s location technology, Pilgrim (*11*), which leverages a wide variety of mobile device signals to pinpoint the time, duration, and location of user visits to locations such as shops, parks, or universities. Unlike the population-level mobility trends published by Google from cell phone geolocation data (*12*), the data are disaggregated by age. User venue visits were aggregated and projected to estimate for each state and two metropolitan areas daily percent changes in venue visits for individuals aged 18−24, 25−34, 35−44, 45−54, 55−64, and 65+ years relative to the the baseline period February 3 - February 9, 2020 (figs. S1 and S2, and supplementary materials).

**Fig. 1:**
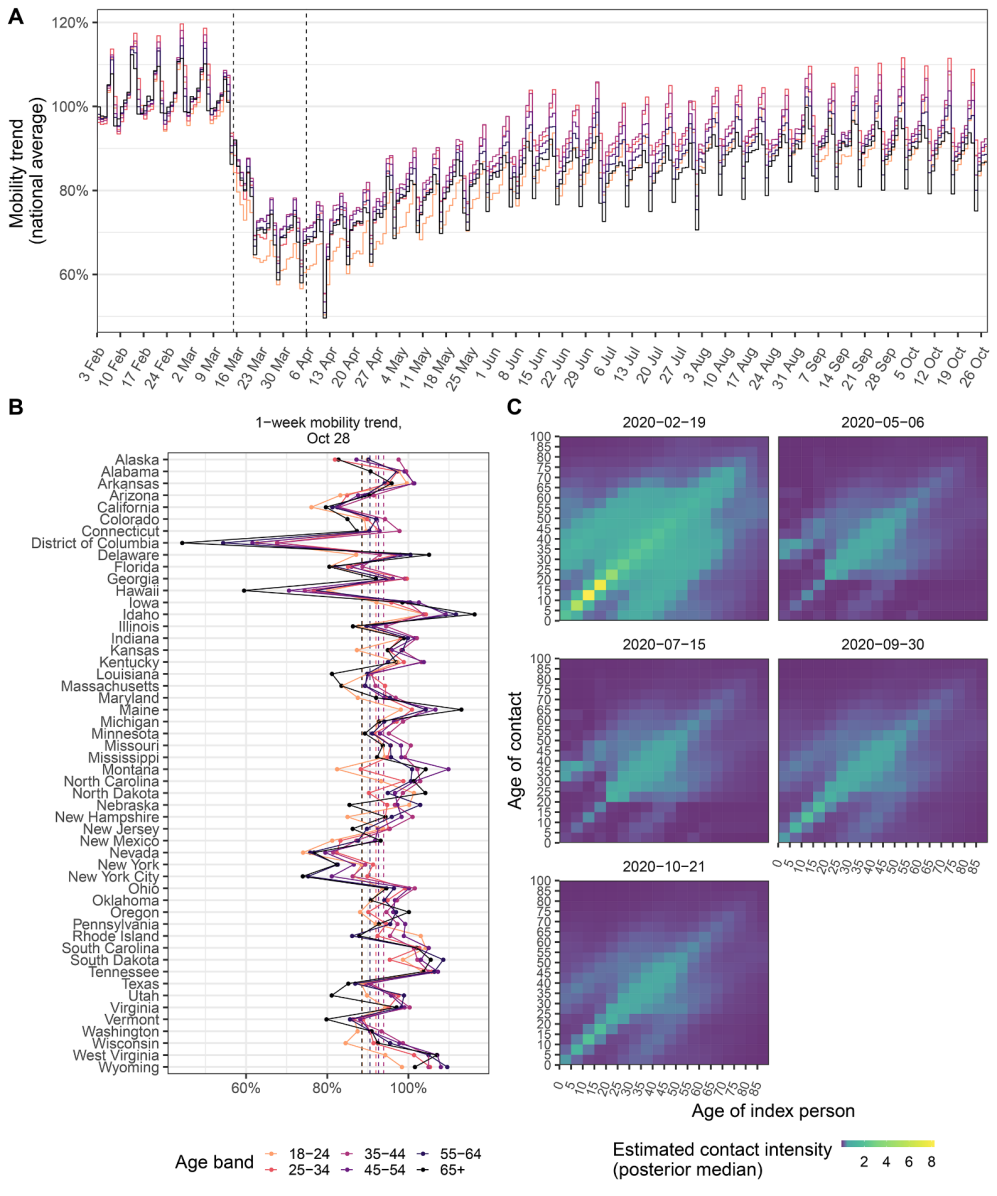
Mobility trends, and estimated time evolution of contact intensities in the United States. (**A**) National, longitudinal mobility trends for individuals aged 18-24, 25-34, 35-44, 45-54, 55-64, 65+, relative to the baseline period February 3 to February 9, 2020. Projected per capita visits standardised daily visit volumes by the population size in each location and age group. The vertical dashed lines show the dip and rebound dates since when mobility trends began to decrease and increase, which were estimated from the time series data. (**B**) 1-week average of age-specific mobility trends between October 22, 2020 - October 28, 2020 across the United States. (**C**) Inferred time evolution of contact intensities in California, calculated as per Eq. 4.

Across the US as a whole, the mobility trends indicate substantial initial declines in venue visits followed by a subsequent rebound for all age groups (Fig. 1A and fig. S1). During the initial phase of epidemic spread, trends declined most strongly among individuals aged 18-24 years across almost all states and metropolitan areas, and subsequently tended to increase most strongly among individuals aged 18-24 in the majority of states and metropolitan areas (fig. S3), consistent with re-opening policies for restaurants, night clubs, and other venues (*10*, *13*, *14*). Yet, considering both the initial decline and subsequent rebound until October 28, 2020, our data indicate that mobility levels among individuals aged *<*35 years have not increased above those observed among older individuals (Fig. 1B and fig. S3).

Mobile phone signals are challenging to analyze, owing e.g., to daily fluctuations in the user panel providing location data, imprecise geolocation measurements, and changing user behavior (*15*). We cross-validated the inferred mobility trends against age-specific mobility data from a second mobile phone intelligence provider, Emodo. This second data set quantified the daily proportions of age-stratified users who spent time outside their home location, and also showed no evidence for faster mobility rebounds among young adults aged *<*35 years as compared to older age groups (see supplementary materials). While other age-specific behavioral differences in for example consistent social distancing, mask use, duration of visits, or types of venues visited could also explain age-specific differences in transmission risk (*10*, *13*, *14*, *16*, *17*), these observations nonetheless led us to hypothesize that the resurgent epidemics in the US may not be driven by increased transmission from young adults aged 20-34.

## Reconstructing human contact patterns and SARS-CoV-2 transmission

To test this hypothesis and disentangle the various factors, we incorporated the mobility data into a Bayesian contact-and-infection model that describes time-changing contact and transmission dynamics at state and metropolitan area-level across the US. For the time period prior to changes in mobility trends, we used data from pre-COVID-19 contact surveys (*6*), and each locations’s age composition and population density to predict contact intensities between individuals grouped in 5-year age bands (figs. S4 to S6), similar as in (*18*). On weekends, contact intensities between school-aged children and teens are lower than on weekdays, while intergenerational contact intensities are higher. In the model, the observed age-specific mobility trends of Fig. 1 are then used to estimate in each location (state or metropolitan area) daily changes in age-specific contact intensities for individuals aged 20 and above. For younger individuals, for who mobility trends are not recorded, contact intensities during school closure periods were set to estimates from two contact surveys conducted post COVID-19 emergence (*7*, *8*). After school reopening in August 2020, relative changes in disease relevant contacts from and to children and teens aged 0-19 were estimated through the model. Contact intensities between children and teens were modeled and estimated separately, to account for potentially lower or higher disease relevant contacts between children and teens in the context of existing non-pharmaceutical interventions within and outside schools (see Materials and methods). As in (*19*), the model further incorporates random effects in space, time, and by age to allow for unobserved, potential age-specific factors that could modulate disease-relevant contact patterns. These random effects enabled us to identify signatures of age-specific, behavioral drivers of SARS-CoV-2 transmission beyond the mobility data in Fig. 1, that may underlie the highly heterogeneous epidemic trajectories across the US. Finally, the reconstructed contact intensities are used in the model to estimate the rate of SARS-CoV-2 transmission, and subsequently infections and deaths. Figure 0 in the extended abstract provides a model overview, and full details are in the supplementary materials.

## Estimated disease dynamics closely reproduce age-specific COVID-19 attributable death counts

The contact-and-infection model was fitted to the Foursquare mobility trends, and age-specific, COVID-19-attributed mortality time series data, which we recorded daily from publicly available sources in 42 US states, the District of Columbia and New York City since March 15, 2020 (fig. S7, see also supplementary materials). Our overall rationale was that, reflecting the highly structured nature of human contacts, transmissions from age groups are received by specific other age groups, and mortality accrues in the age groups receiving infections. Thus, working back from the time evolution of reliably documented, age-specific COVID-19 attributable deaths, it is possible to reconstruct age-specific drivers of transmission during particular periods in time. Inference was performed in a Bayesian framework and restricted to 38 US states, the District of Columbia and New York City with at least 300 COVID-19-attributed deaths, giving a total of 8,676 observation days. The estimated disease dynamics closely reproduced the age-specific COVID-19 death counts (fig. S8).

**Fig. 2:**
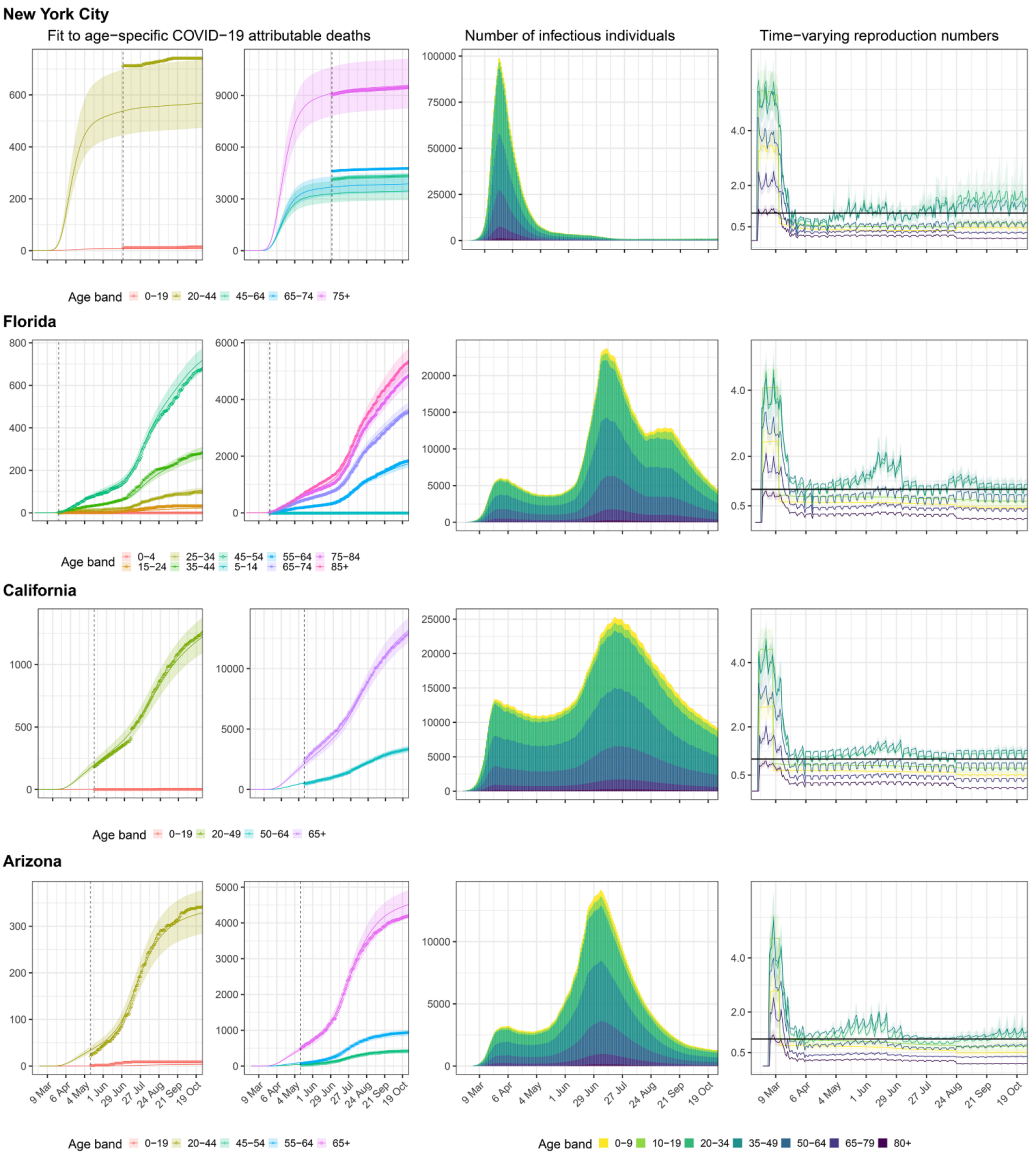
Model fits and key generated quantities for New York City, California, Florida and Arizona. (**Left**) Observed cumulative COVID-19 mortality data (dots) versus posterior median estimates (line) and 95% credible intervals (ribbon). The vertical line indicates the collection start date of age-specific death counts. (**Middle**) Estimated number of infectious individuals by age (posterior median). (**Right**) Estimated age-specific effective reproduction number, posterior median (line) and 95% credible intervals (ribbon).

Figure 2 illustrates the model fits for New York City, Florida, California, and Arizona, showing that the inferred epidemic dynamics differed markedly across locations. For example, in New York City, the epidemic accelerated for at least 4 weeks since the 10th cumulative death and until age-specific reproduction numbers started to decline, resulting in an epidemic of large magnitude as shown through the estimated number of infectious individuals (Fig. 2, mid column). Subsequently, we find that reproduction numbers for all age groups were controlled to well below one except for individuals aged 20-49 (Fig. 2, rightmost column), resulting in a steady decline of infectious individuals. In the model, children and teens returned to their pre-lockdown contact intensities on August 24, 2020 or later, depending on when state administrations no longer mandated state-wide school closures, and relative decreases or increases in their disease relevant contact intensities after school-reopening were estimated. Concomitantly, reproduction numbers from children aged 0-9 and teens aged 10-19 increased, but as of the last observation week in October 2020 we find no strong evidence that their reproduction numbers have exceeded one at population level in most states and metropolitan areas considered. Detailed situation analyses for all locations are presented in the supplementary materials.

## SARS-CoV-2 transmission is sustained primarily from age groups 20-49

**Fig. 3:**
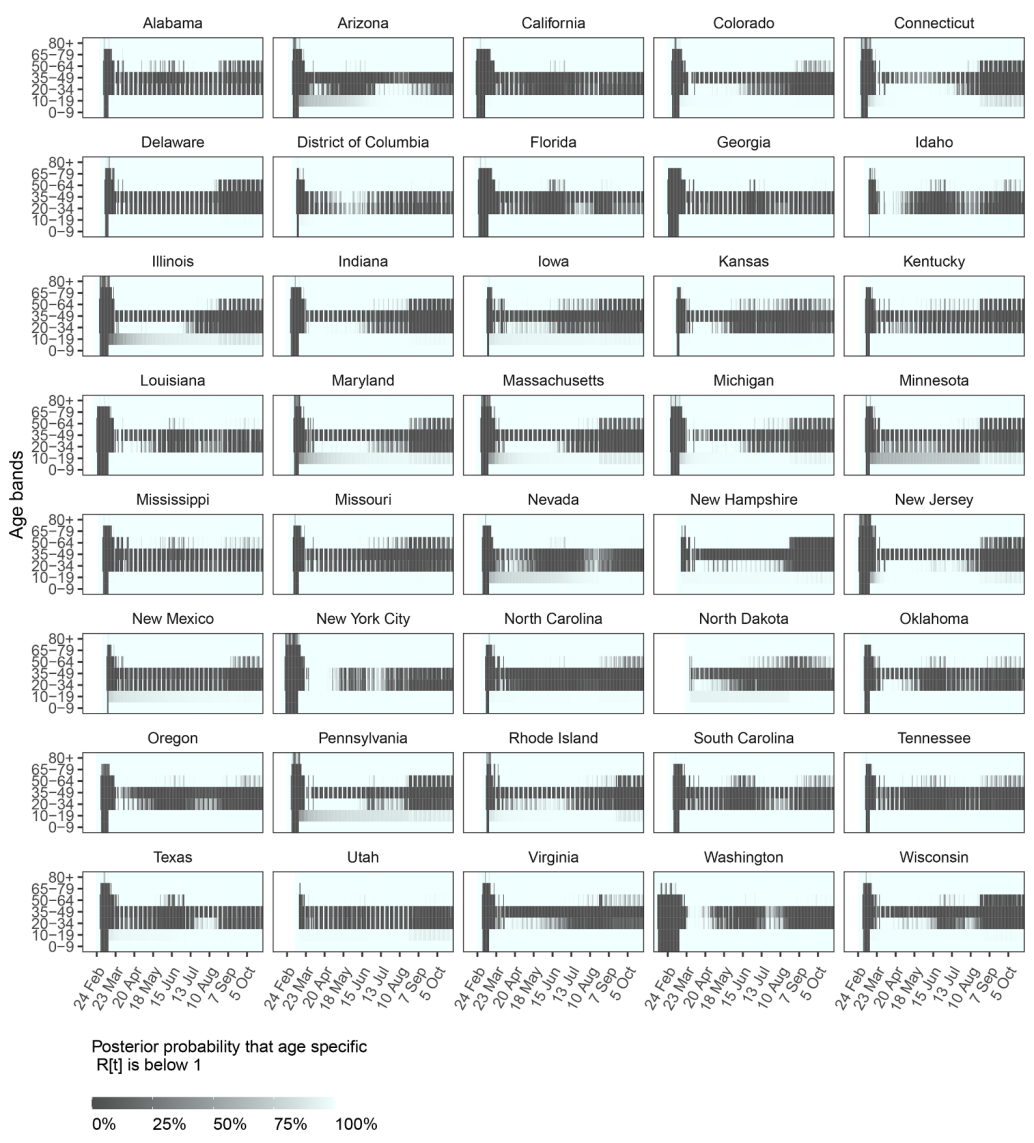
Time evolution of estimated age-specific SARS-CoV-2 reproduction numbers across the US. Each panel shows for the corresponding location (state or metropolitan area) the estimated posterior probability that the daily effective reproduction number from individuals stratified in 7 age groups were below one. Darker colors indicate low probability that reproduction numbers were below one.

Figure 3 summarizes the epidemic situation for all states and metropolitan areas evaluated, and the age groups that sustain COVID-19 spread. In the last observation week in October 2020, the estimated reproduction number across all locations evaluated was highest from individuals aged 35-49 (1.39 [1.34-1.44]) and 20-34 (1.29 [1.24-1.36]), and around one for age groups 1019 and 50-64 (tables S1 and S2). These trends across age groups were largely consistent over time. The primary mechanisms underlying the high reproduction numbers from 20–49-year-olds are that at population level, adults aged 20-49 naturally have most contacts to other adults aged 20 and above, which are more susceptible to COVID-19 than younger individuals, paired with increasing mobility trends for these age groups since April 2020 (Fig. 1 and fig. S6). In addition, from the death time series data, the model inferred characteristic random effect signatures in time and by age across locations (fig. S9), which indicate elevated transmission risk per venue visit for individuals aged 20-49 relative to other age groups. Figure S10 visualizes the combined, estimated effects of mobility and behavior on transmission risk, and reveals together with Fig. 3 considerable heterogeneity in age-specific transmission dynamics across locations. While the model consistently estimates effective reproduction numbers close to or above one across all locations from adults aged 35-49, disease dynamics are more variable from young adults aged 20-34, with some states (Arizona, Florida, Texas) showing sustained transmission from young adults in May and June, and other states (e.g., Colorado, Illinois, Wisconsin) showing sustained transmission from young adults since August. This suggests that additional interventions to adults aged 20-49, including rapid mass vaccination if vaccines prove to block transmission, could bring resurgent COVID-19 epidemics under control.

## The majority of COVID-19 infections originate from age groups 20-49

**Fig. 4:**
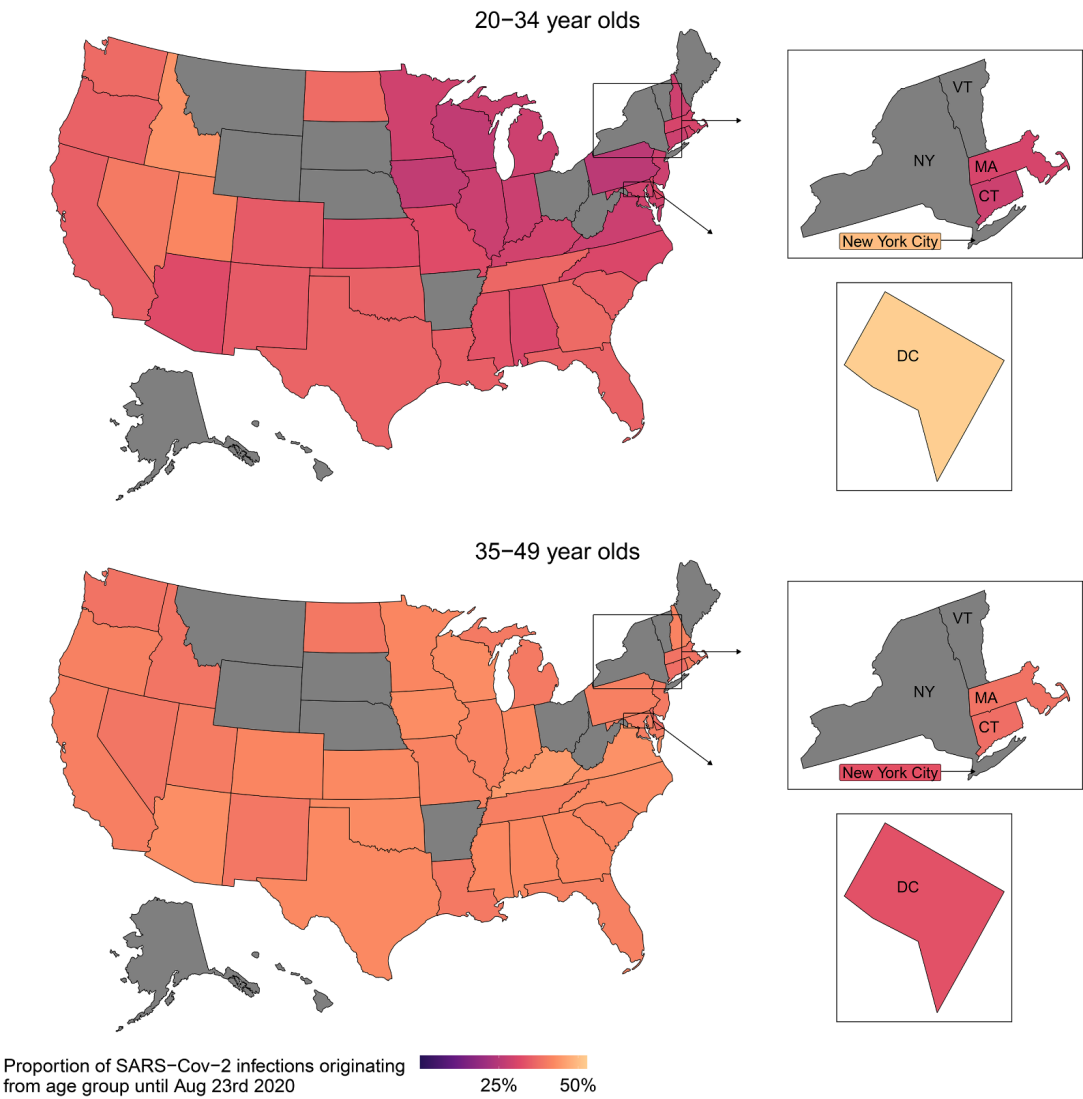
Estimated spatial variation in the share of young adults aged 20-34 and adults aged 35-49 to COVID-19 spread until mid August, 2020. Posterior median estimates of the contribution to cumulated SARS-CoV-2 infections until August 17, 2020, prior to school reopening in the first states in the model. State-level COVID-19 epidemics not considered in this study are in grey.

To quantify how age groups contribute to resurgent COVID-19, it is not enough to estimate reproduction numbers, because reproduction numbers estimate the number of secondary infections per infectious individual, and the number of infectious individuals varies by age as a result of age-specific susceptibility gradients and age-specific contact exposures. We therefore considered the reconstructed transmission flows and calculated from the fitted model the contribution of each age group to new infections in each US location over time. Across all locations evaluated, we estimate that until mid-August 2020, before schools were considered to re-open in the first locations in the model, the percent contribution to onward spread was 41.1% [40.7%-41.4%] from individuals aged 35-49, compared to 2.1% [1.6%-2.8%] from individuals aged 0-9, 4.0% [3.5%-4.6%] from individuals aged 10-19, 34.7% [33.9%-35.5%] from individuals aged 20-34, 15.3% [14.8%-15.8%] from individuals aged 50-64, 2.5% [2.2%-2.9%] from individuals aged 65-79 age group, and 0.3% [0.3%-0.3%] from individuals aged 80+ (table S4). Spatially, the contribution of adults aged 35-49 were estimated to be remarkably homogeneous across states, whereas the estimated contributions of young adults aged 20-34 to COVID-19 spread tended to be higher in Southern, South-western, and Western regions of the US (Fig. 4), in line with previous observations (*4*).

## No substantial shifts in age-specific disease dynamics over time

**Fig. 5:**
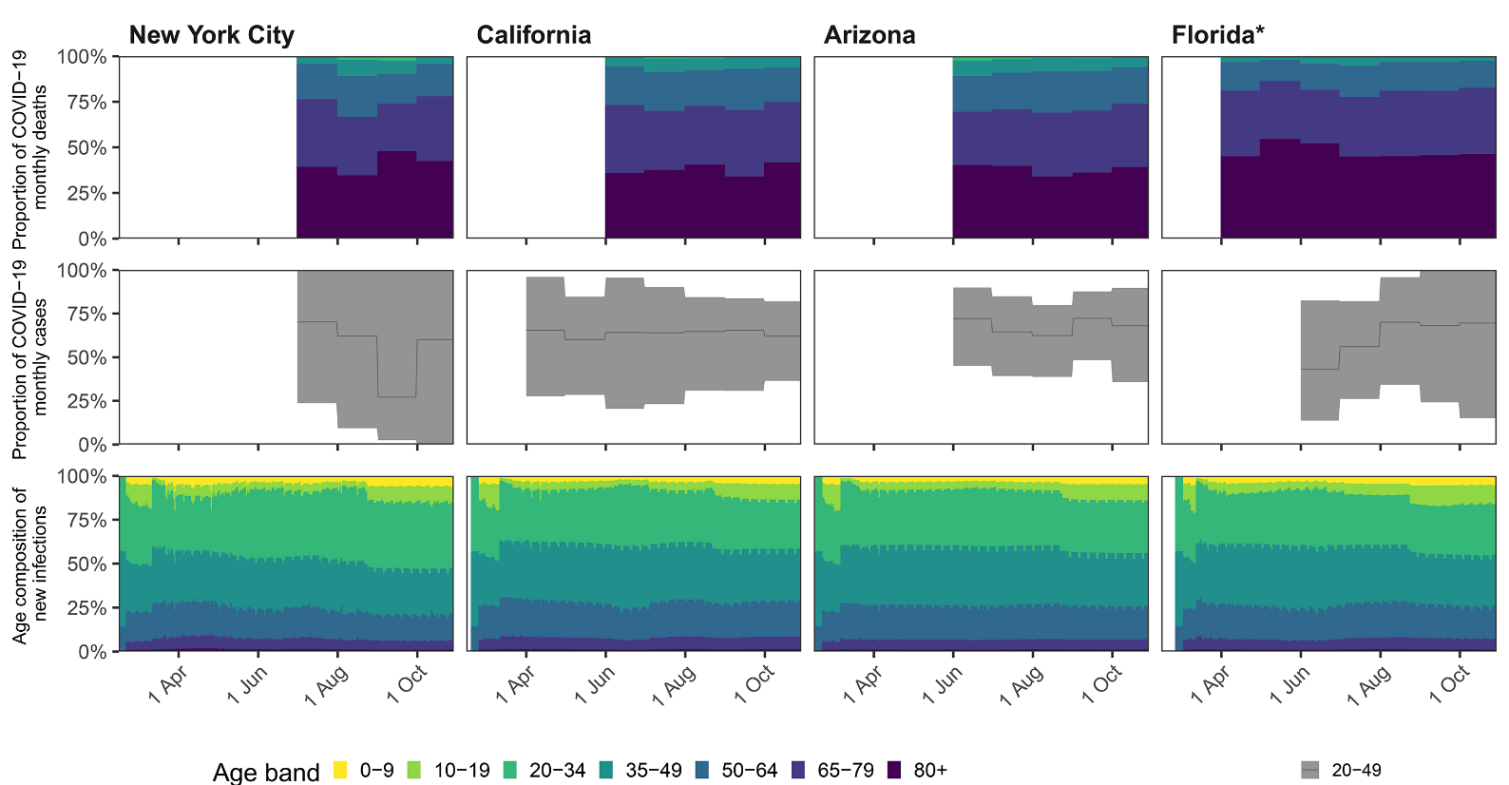
Share of age groups among COVID-19 attributable deaths and infections in the United States. (**Top**) Proportion of monthly observed deaths attributed to COVID-19 by age group. Age-specific COVID-19 attributable deaths were recorded from state or city Departments of Health. Departments of Health used their own age stratification, and the observed data were re-estimated into common age groups across states with a Dirichlet-Multinomial model (see supplementary materials). A star (^∗^) next to a location’s name indicates that there was a statistically significant shift in the share of individuals aged 80+ among deaths in the corresponding location. (**Middle**) Proportion of monthly reported cases among 20-49 year olds. Monthly cases were back-calculated using the meta-analysis infection fatality rate estimates of (*20*). The figure shows the estimated share of individuals aged 20-49 among monthly cases (posterior median: line, 95% credible interval: ribbon). (**Bottom**) New daily estimated infections by age group for New York City, Florida, California and Arizona (posterior median).

Over time, we found that the share of age groups among the observed COVID-19 attributable deaths was remarkably constant (Fig. 5A and fig. S11), which stands in contrast to the large fluctuations in the share of age groups among reported cases (*4*). To test for shifts in the share of age groups among COVID-19 infections, we next back-calculated the number of expected, age specific infections per calendar month of aggregated COVID-19 attributable deaths using meta-analysis estimates of the age-specific COVID-19 infection fatality ratio (*20*). This empirical analysis suggested no statistically significant trends in the share of age groups among COVID19 infections (Fig. 5B and fig. S12), which is further supported by model estimates (Fig. 5C and fig. S13). Based on the combined mobility and death data, we find the reconstructed fluctuations in age-specific reproduction numbers had only a relatively modest impact on the contribution of age groups to onward spread over time, and no evidence that young adults aged 20-34 were the primary source of resurgent COVID-19 in the US over summer 2020. These results underscore that, when testing rates are heterogeneous and not population representative, it is challenging to determine the age-specific pattern of transmission based only on reported case data.

## School reopening has not resulted in substantial increases in COVID-19 attributable deaths

Between August and October 2020, school closure mandates have been lifted in 39 out of 40 of the US locations evaluated in this study, and provided 2,570 observation days to estimate the impact of school reopening on COVID-19 spread. The following analyses are therefore based on fewer data points than those aforementioned, rely on mortality figures accrued until end of October 2020, as well as reported school case data from Florida and Texas, which were used to define lower and upper bounds on cumulative attack rates among children and teens aged 5-18 (see Materials and methods). Reflecting stuttering transmission chains in school settings, reproduction numbers from children aged 0-9 and teens aged 10-19 were estimated at below one (respectively 0.52 [0.42-0.60] and 0.73 [0.57-0.88]) after schools were considered to have reopened in the model (Fig. 3 and table S2). Reproduction numbers from children were lower than from teens because at population-level preschoolers have fewer contacts than school-aged children (fig. S6).

**Fig. 6:**
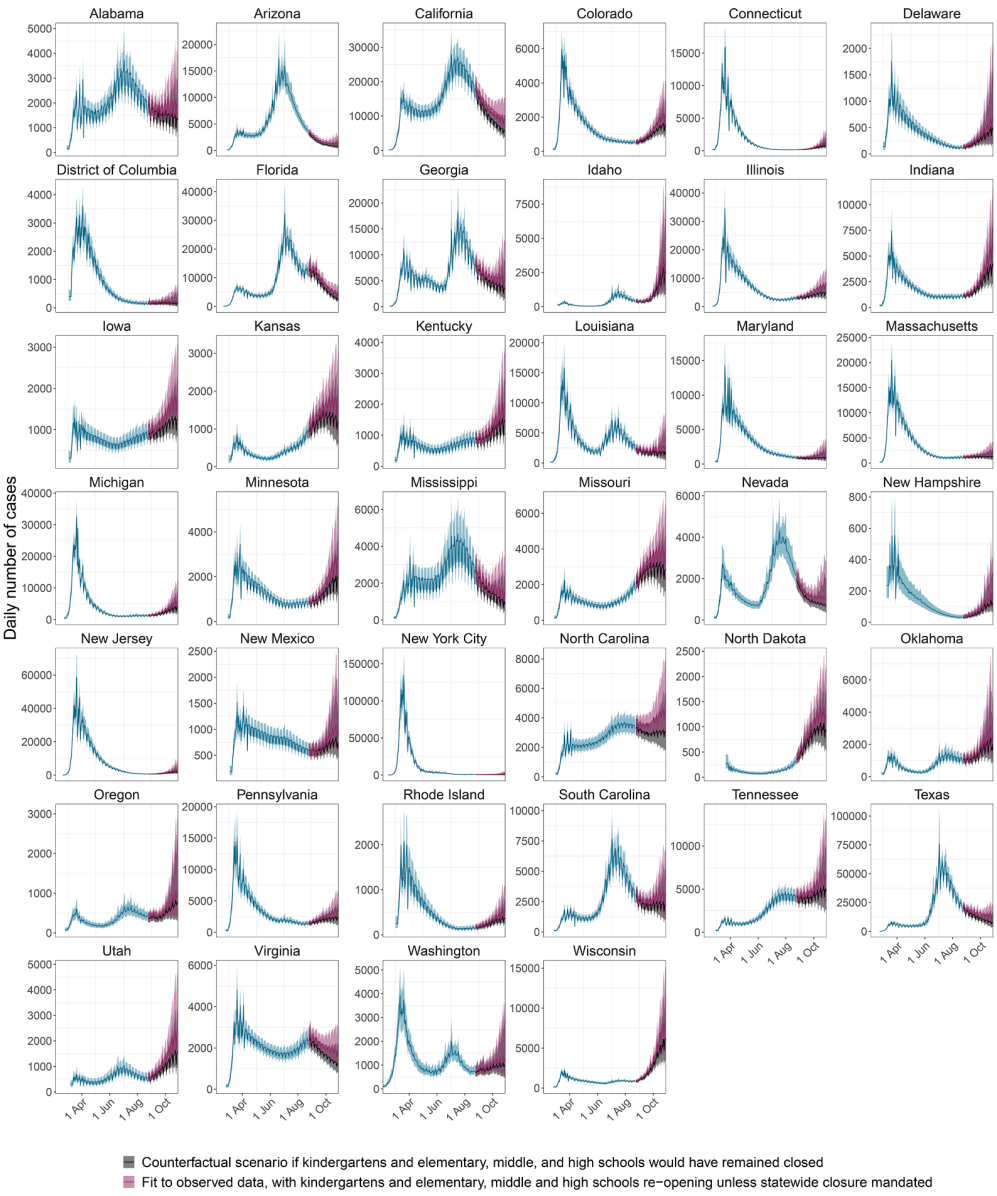
**Retrospective counterfactual modelling scenarios exploring the impact of school reopening on COVID-19-attributable cases**. Shown in blue and red are estimated, daily COVID-19 cases (posterior median: line, 95% credible interval: ribbon) under the model until October 29, 2020, assuming reported cases among school-aged children from Florida and Texas under-report actual cases by a factor of 6 or less. In counterfactual modelling scenarios, the retrospective impact of continued school closures was explored until October 29, 2020, and the predicted case trajectories are shown (posterior median: black line, 95% credible interval: black ribbon).

Since school closure mandates were lifted, the higher reproduction numbers from children and teens resulted in age shifts in the sources of SARS-CoV-2 infections. In October 2020 an estimated 2.7% [1.8%-3.7%] of infections originated from children aged 0-9, 7.1% [4.5%-10.3%] from teens aged 10-19, 34.0% [31.9%-36.4%] from 20-34, 38.2% [36.7%-39.4%] from 35-49, 15.1% [14.1%-16.1%] from 50-64, 2.5% [2.2%-2.9%] from 65-79, and 0.3% [0.2%-0.3%] from individuals aged 80+ across all locations evaluated (table S5 vs table S4). The reconstructed shifts in the age of COVID-19 sources after school reopening are relatively modest compared to the typical age profile of infection sources of pandemic flu (*21*), and reflect lower age-specific susceptibility to SARS-CoV-2 transmission among children and teens, but also substantially fewer, inferred disease relevant contacts from children and teens than would be expected from their corresponding pre-pandemic contact intensities. The mechanisms behind these beneficial effects remain unclear, but the model suggests they are substantial. In retrospective counterfactual scenarios we explored what COVID-19 case and death trajectories would have been expected if schools had remained closed, and find a large overlap between the counterfactual and actual case and death trajectories (Fig. 6, fig. S15). However, since children and teens seed infections in older age groups that are more transmission efficient, as of October 2020, school opening is associated with an estimated 25.7% [14.5%-40.5%] increase of COVID-19 infections and a 5.9% [3.4%-9.3%] increase in COVID-19 attributable deaths (table S7). Larger proportions of COVID-19 infections and deaths are attributed to school re-openings if the actual number of cases among school-aged children is more than six times larger than the number in school situation reports (table S7). These findings indicate that adults aged 20-34 and 3549 continue to be the only age groups that contribute disproportionally to COVID-19 spread relative to their size in the population (fig. S14), and that the impact of school reopening on resurgent COVID-19 is mitigated most effectively by strengthening disease control to adults aged 20-49.

## Caveats

The findings of this study need to be considered in the context of the following limitations. Rossen and colleagues (*22*) observed that US excess deaths between the beginning of the pandemic and October 2020 were by 38% higher than the reported COVID-19 attributable deaths, suggesting that the death data on which this analysis rests are subject to under-reporting. The scale of the US epidemics may be larger than we infer, and our age-specific analyses may be biased if underreporting of deaths depends on age. However, due to the high proportion of asymptomatic COVID-19 cases (*5*), underreporting is a substantially larger caveat for reported case data, and in particular the observed shifts in the share of age groups among reported cases (*4*, *23*), which are absent from the share of age groups among reported deaths (fig. S11). This suggests that age-specific death data provide a more reliable picture into resurgent COVID-19 epidemics than reported cases. We further rely on limited data from two contact surveys performed in the United Kingdom and China to characterize contact patterns from and to younger individuals during school closure periods (*7*, *8*), and this could have biased our findings that children and teens have contributed negligibly to SARS-CoV-2 spread until school reopening. To address this limitation, we explored the impact of higher inter-generational contact intensities involving children during school closure periods, and in these analyses the estimated contribution of children aged 0-9 to onward spread until August 2020 remained below 5% and the contribution of teens aged 10-19 remained below 12.5% (see supplementary materials). Epidemiologic models are sensitive to assumptions on the infection fatality ratio (IFR) that enables the estimation of actual cases from observed deaths by age. Our analyses are based on a meta-analysis that consolidates estimates from 27 studies and 34 geographic locations (*20*). To test the assumed IFR, we compared the scale of the estimated resurgent epidemics against data from sero-prevalence surveys conducted by the Centers for Disease Control and Prevention (CDC) (*24*), and found good congruence (table S6 and supplementary materials). The COVID19 epidemic is more granular than considered in our spatial modelling approach. Substantial heterogeneity in disease transmission exists at county level (*25*), and our situation analyses by state and metropolitan areas need to be interpreted as averages. To no exception, the model underlying our analyses also relies on simplifying mathematical assumptions on population-level disease spread, which may be shown unsuitable as further evidence on SARS-CoV-2 transmission accumulates (*26*). For instance, the model assumes children and teens are as transmissible as adults, which has been challenging to quantify to date (*27*), and falls short of accounting for population structure other than age, such as household settings, where attack rates have been estimated to be substantially higher than in non-household settings (*28*). It is possible that the model under-estimates the impact of school reopening on SARS-CoV-2 transmission.

Data from countries that have re-opened schools have provided little evidence for substantial transmission in schools, nor significantly increased community-level infection rates after school reopening until the emergence of more transmissible SARS-CoV-2 variants (*29*, *30*), but this might reflect frequent sub-clinical infection among school-aged children. More transmissible SARS-CoV-2 variants could increase reproduction numbers to above one for all age groups, which implies substantial spread from all age groups, and require generally stricter control measures across all ages to prevent COVID-19 attributable deaths (*31*).

## Conclusions

This study provides evidence that the resurgent COVID-19 epidemics in the US in 2020 have been driven by adults aged 20-49, and in particular adults aged 35-49, before and after school reopening. Unlike pandemic flu, these adults accounted after school reopening in October, 2020 for an estimated 72.2% [68.6%-75.9%] of SARS-CoV-2 infections in the US locations considered, whereas less than 5% originated from children aged 0-9 and less than 10% from teens aged 10-19. The population mobility data, and the death data provided by state and city Departments of Health reveal heterogeneous disease spread in the US, with higher transmission risk per venue visit attributed to individuals aged 20-49 over distinct time periods, and younger epidemics with a greater share of individuals aged 20-34 among cumulated infections in the South, South-western, and Western regions of the US. Over time, the share of age groups among reported deaths has been remarkably constant, suggesting that young adults are unlikely to have been the primary source of resurgent epidemics since summer 2020, and that instead changes in mobility and behavior among the broader group of adults aged 20-49 underlie resurgent COVID-19 in the US in 2020. This study indicates that in locations where novel highly-transmissible SARS-CoV-2 lineages have not yet established, additional interventions among adults aged 20-49, such as mass vaccination with transmission-blocking vaccines, could bring resurgent COVID-19 epidemics under control and avert deaths.

## Materials and Methods

To characterize the role of age groups in driving resurgent COVID-19, we have taken a systematic approach that involved data collection, mathematical modelling, likelihood-based inference, and validation against external data. The following sections summarize our materials and methods, and full technical details are in the Data Availability Statement and the supplementary Materials.

### Data and data processing

The analyses presented in this study are based on age-specific COVID-19 attributable mortality counts that were collected daily from US state and city Departments of Health (DoH), all-age COVID-19 death counts, all-age COVID-19 case counts, COVID-19 case counts in school settings K1-K15, human contact data before and during the pandemic, and human mobility data during the pandemic.

Briefly, age-specific COVID-19 cumulative death counts were retrieved for 42 US states, the District of Columbia and New York City from city or state DoH websites, data repositories, or via data requests to DoH (table S8). Data were checked for consistency and adjusted when necessary. Age-specific COVID-19 death time series were reconstructed from cumulative counts, and the time series were used for model fitting (*32*).

All-age daily COVID-19 case and death counts from February 01, 2020 until October 30, 2020 regardless of age were obtained from John Hopkins University (JHU) for all U.S. states and the District of Columbia (*3*), except New York State. For New York State, daily COVID-19 death counts from February 01, 2020 until October 30, 2020 were obtained from the New York Times’ (NYT) data (*33*). For New York City, daily COVID-19 deaths counts were obtained from the GitHub Repository (*34*). The all-age death counts were used for model fitting prior to when age-specific death counts were reported for each location, and all-age case counts were used for model fitting for the entire study period.

COVID-19 case counts in school settings K1-K15 were retrieved for Florida and Texas and matched with student enrolment numbers in each school from the Common Core of Data Americas Public Schools database (*35*). Cumulative attack rates were obtained by dividing cumulative reported cases among students by student numbers, and used for model fitting.

Human contact data before the pandemic were obtained from the Polymod study (*6*), and used to predict baseline contact matrices during the early part of the pandemic for each location, similar as in (*18*). Given the variation in contact patterns seen across survey settings, baseline contact matrices for each study location in the US were predicted based on each location’s population density and age composition with a log linear regression model. Age-specific population counts were obtained from (*36*). Area measurements were obtained for every US states and for New York City respectively from (*37*) and (*38*). Contact matrices were predicted by 5-year age bands for weekdays and weekends, and used in the model. Human contact data during the pandemic were retrieved from two surveys (*7*, *8*), and used in the model to specify contact patterns from and to individuals aged 0-19 during periods of school closure.

Age-specific human mobility trends were derived from the Foursquare Labs Inc. US first-party panel that includes >10 million of opt-in, always-on active users. From operated and partner apps, Foursquare collect a variety of device signals against opted-in users including intermittent device GPS coordinate pings, WiFi signals, cell signal strength, device model, and operating system version. A smaller set of labeled explicit check-ins are captured from a portion of the user panel. Check-ins are explicit confirmations that a user was at a given venue at a given point of time, and serve as training labels for a non-linear model that is used to predict visits among users with unlabeled visits in terms of probabilities as to which venue users ultimately visited (*11*). Visit probabilities among panellists were processed and aggregated by day, age, and study location, and standardised to daily per capita visits using latest US Census data. Percent changes in daily venue visits by age and study location were obtained relative to the baseline period February 3 to February 9, 2020, and used for analysis and model fitting. For validation purposes, a second mobility data set was obtained from Emodo. The Emodo data set quantifies the proportion of individuals with at least one observed ping outside the user’s home location, out of a panel of individuals whose GPS enabled devices emitted at least one ping on the corresponding day. Primary data were similarly aggregated by day, age, and study location, standardised to daily per capita visits using latest US Census data, and mobility trends were calculated relative to the baseline period February 19 to March 3, 2020.

### Statistical analysis of human mobility data and COVID-19 attributable death data

The age-specific human mobility data showed marked time trends, which were characterised in terms of three phases defined by the dip date after which the 15-day moving average fell below 10% compared to the average value in the two prior weeks, and the rebound date that corresponded to the date at which the 15-day moving average was lowest. Differences in the mobility trends relative to the February baseline period, before and after rebound dates, and relative to individuals aged 35-44 were assessed using Gamma regression models using log link and location by age interaction covariates.

To characterize the time evolution of deaths across locations and validate model fits, age-specific COVID-19 attributable deaths among the same age strata across locations were predicted by month with Dirichlet-Multinomial regression models. Trends in the share of age groups among monthly deaths were assessed by testing for differences in the proportions in the first month relative to subsequent months.

To test for potential differences in age-specific transmission dynamics based on the collected death data and without epidemic models, meta-analysis estimates of age-specific infection fatality ratios (*20*) were used to predict the share of age groups among infections from monthly age-specific deaths. Trends in the share of age groups among monthly infections were assessed by testing for differences in the proportions in the first month relative to subsequent months.

### Contact-and-infection model

To quantify age-specific aspects of COVID-19 spread in heterogeneous populations, we formulated an age-specific, discrete-time renewal model in which disease transmission occurs via contact intensities between population groups stratified by 5-year age bands. The model has four key features described below. First, contact intensities vary in time and are inferred from signatures in the age-specific mortality and mobility data. This feature aims to reflect the substantial changes in human contact patterns during the pandemic (*7*–*9*). Second, the challenge and value of the model to produce generalizable knowledge is to explain disease spread across multiple locations with distinct demographics simultaneously. To this end, the renewal equations were embedded into a hierarchical model in which information on disease spread is borrowed across locations (*1*, *39*). Third, the model describes disease spread during the initial and later phase of the pandemic, as mobility patterns become less correlated with transmission risk and schools reopen (*40*, *41*). This feature allowed us to test for changes in disease dynamics over time. Fourth, the model is fitted in a Bayesian framework to the all-age and age-specific death data, all-age case data, case data from schools, and age-specific human mobility trends (*42*). This feature forced us to focus on a model whose parameters are inferable from the data across all locations. The model is described in detail in the supplementary materials.

Briefly, we consider populations stratified by the 5-year age bands A, such that

a ∈ A = {[0-4], [5-9], …, [75-79], [80-84], [85+]}(1)

and denote the number of new infections, *c*, on day *t*, in age band *a*, and location *m* as $c_{m,t,a}$. In the renewal equation, past infections are weighted by their relative infectiousness on day *t*, and the sum of these individuals has contacts with individuals in other age groups. Contacts are described by the expected number of disease relevant human contacts one person in age *a* has with other individuals in age band *a’* on day *t* in location *m*, $c_{m,t,a,a^{\prime }}$. Upon contact, a proportion $s_{m,t,a^{\prime }}$of individuals of age *a’* on day *t* in location *m* remains susceptible to SARS-CoV-2 infection, and transmission occurs with probability $\rho_{a^{'}}$. Thus, the age-specific renewal equation with time-changing contact intensities is

$$c_{m,t,a^{\prime }}=s_{m,t,a^{\prime }}\rho_{a^{\prime }}\underset{a}{\sum }C_{m,t,a,a^{\prime }}\left(\overset{t-1}{\underset{s=1}{\sum }}c_{m,s,a}\;g\left(t-s\right)\right)$$ (2)

where *g* quantifies the relative infectiousness of individuals *s* days after infection. An important feature of SARSCoV-2 transmission is that similarly to other coronaviruses but unlike pandemic influenza (*43*), susceptibility to SARS-CoV-2 infection increases with age (*7*, *21*, *44*). Here, we used contact tracing data from Hunan province, China (*7*) to specify lower susceptibility to SARS-CoV-2 infection among children aged 0-9, and higher susceptibility among individuals aged 60+, when compared to the 10-59 age group as part of the transmission probabilities ${\text{ρ}}_{a^{\prime }}$. Previously infected individuals are assumed to be immune to re-infection within the analysis period, consistent with mounting evidence for sustained antibody responses to SARS-CoV-2 antigens (*45*, *46*), so that

$$s_{m,t,a^{\prime }}=1-\frac{\sum_{s=1}^{t-1}c_{m,t,a^{\prime }}}{N_{m,a^{\prime }}},$$ (3)

where $N_{m,a^{\prime }}$ denotes the population count in age group *a’* and location *m*.

For adults aged 20+, the time changing contact intensities were described in terms of the pre-pandemic baseline contact intensities in location *m*, which we denote by $C_{m,t,a,a^{\prime }}$, and expected reductions in disease relevant contacts from contacting individuals of age *a* on day *t* in location *m*, which we denote by ${\text{η}}_{m,t,a}$, and contacted individuals of age *a’* on day *t* in location *m*, ${\text{η}}_{m,t,a^{\prime }}$,

$$C_{m,t,a,a^{\prime }}={\text{η}}_{m,t,a}\;C_{m,a,a^{\prime }}\;{\text{η}}_{m,t,a^{\prime }},$$ (4)

where *a*, *a’* ∈ {[20−24]*, ...,* [85+]}. Expected reductions in disease relevant contacts were specified as a random effects model that included the observed, age-specific mobility trends as covariates. In the model, each age-specific mobility trend was decoupled into three separate covariates that reflect the initial pre-pandemic, dip, and rebound phases in human mobility trends, so that previously observed decreases in correlation between mobility trends and transmission risk could be captured (*40*, *41*, *47*). As the same number of venue visits in e.g., Wyoming may translate to different transmission risk than in e.g., New York City, spatial random effects allowed for scaling of mobility trends during the dip and rebound phase in each location. As venue visits do not capture all aspects of transmission risk, the model further incorporates independently for each location autocorrelated biweekly random effects to capture information on elevated, disease relevant contact intensities and transmission risk that is present in the death time series data. To test for age-specific signatures of elevated transmission risk, the model further included for each location age-specific random effects for individuals aged 20-49.

For children and teens aged 0-20, mobility data are not available, and during periods of school closure the contact intensities from and to children and teens were set to the average contact intensities reported in (*7*). This implied that relative to pre-pandemic contact patterns, peer-based contacts were substantially reduced, whereas contacts from an adult to children and teens increased slightly. In the model, schools were set to re-open on or after August 24, 2020 when state administrations no longer mandated state-wide school closures by that date (*48*, *49*). Thereafter, Eq. 4 was extended to include children and teens, and expected mobility reductions were estimated from the case and death data. In the absence of further data, a common average effect could be estimated across locations and children and teen age groups for the last two observation months, ${\text{η}}_{m,t,a}={\text{η}}^{\text{children}}$ for *a* ∈ [0 − 20]. A further compound effect *γ* was added to modulate the number of disease relevant child/teenchild/teen contacts, which we interpreted as reduced infectiousness from children and teens and/or a positive impact of non-pharmaceutical interventions among school-aged children and teens.

### Bayesian inference

Past age-specific disease dynamics across all locations were inferred from age-specific death data available across locations, and age-specific mobility data. To do this, in the model, a proportion ${\text{π}}_{m,a}$ of new infections in location *m* of age *a* die, and the day of death is determined by the infection-to-death distribution, which was assumed to be constant across age groups. The proportions ${\text{π}}_{m,a}$ were associated with a strongly informative prior derived from the meta-analysis of (*20*), but were allowed to deviate from the baseline infection fatality ratio through location-specific random effects. The expected number of deaths in location *m* on day *t* in age band *a*, $d_{m,t,a}$, were aggregated to the reporting strata in each location, and fitted to the observed data using a Negative Binomial likelihood model. When age-specific death data were not available, the model was fitted to all-age death data with a Negative Binomial likelihood model. All-age case data were smoothed, and used to specify a lower bound on the overall number of infections $c_{m,t}=\underset{a}{\sum }c_{m,t,a}$ through a student-t cumulative density likelihood model. Case data from schools were used to calculate empirical attack rates in school settings during specified observation windows. In turn, the empirical attack rates were used to describe a lower bound on the actual attack rate among 5-18 year old children and teens in the same observation periods in the model, using a normal cumulative density likelihood model. An upper bound on the actual attack rates was also specified by assuming that actual cases in school settings were under-reported at most 10-fold, using a normal complementary cumulative density likelihood model. The contact-and-infection model was fit with CmdStan release 2.23.0 (22 April 2020), using an adaptive Hamiltonian Monte Carlo (HMC) sampler (*42*). 8 HMC chains were run in parallel for 1*,*000 iterations, of which the first 400 iterations were specified as warm-up. There were no divergent transitions.

### Generated quantities

Results were reported in the age bands *d* ∈ D = {[0−9]*,*[10−19]*,*[20−34]*,*[35−49]*,*[50−64]*,*[65−79]*,*[80+]}.The primary model outputs were aggregated correspondingly, e.g. the number of new infections in location *m* on day *t* in reporting age band *d* was $c_{m,t,d}=\underset{a\in d}{\sum }c_{m,t,a}$. The effective number of infectious individuals *c*^∗^ in location *m* and age band *d* on day *t* was calculated based on the renewal model (*2*), $c_{m,t,d}^{*}=\overset{t-1}{\underset{s=1}{\sum }}c_{m,s,d}\;g\left(t-s\right)$, and is shown in Fig. 2. Following (*2*), the time-varying reproduction number on day *t* from one infectious person in *a* in location *m* is $R_{m,t,a}=\underset{a^{\prime }}{\sum }s_{m,t,a^{\prime }}\;\rho_{a^{\prime }}\;C_{m,t,a,a^{\prime }}$, and the reproduction numbers were aggregated to the reporting strata based on the identity $R_{m,t,d}=\underset{a\in d}{\sum }\left(c_{m,t,a}^{*}\right)/\left(\underset{k\in d}{\sum }c_{m,t,k}^{*}\right)R_{m,t,a}$, and are shown in Fig. 2 and tables S1 and S2. The transmission flows from age group *a* to age group *a’* at time *t* in location *m* are given by $F_{m,t,a,a^{\prime }}=s_{m,t,a^{\prime }}\;\rho_{a^{\prime }}\;C_{m,t,a,a^{\prime }}\;\left(\overset{t-1}{\underset{s=1}{\sum }}c_{m,s,a}\;g\left(t-s\right)\right)$, and are aggregated using $F_{m,t,d,d^{\prime }}=\underset{a\in d,a^{\prime }\in d^{\prime }}{\sum }F_{m,t,a,a^{\prime }}$. In turn, the contributions of age groups to COVID-19 spread are $S_{m,t,d}=\left(\underset{d^{\prime }}{\sum }F_{m,t,d,d^{\prime }}\right)/\left(\underset{d}{\sum }\underset{d^{\prime }}{\sum }F_{m,t,d,d^{\prime }}\right)$, and are reported in tables S4. Cumulated COVID-19 attack rates were calculated through $A_{m,t,d}=\left(\overset{t}{\underset{s=1}{\sum }}c_{m,s,d}\right)/\left(N_{m,d}\right)$, where $N_{m,d}$ is the number of individuals in location *m* and age band *d*, and are reported in table S6.

### Validation and sensitivity analyses

Reconstructed past transmission dynamics were assessed against external data on the scale of the epidemic from seroprevalence surveys conducted across the US by the CDC (*24*). Validation results are reported in the supplementary materials, suggesting larger discrepancies between model fit and seroprevalence data for Connecticut and New York City, with larger epidemics reconstructed in the model than the data suggest. The contact-and-infection model does not account for sustained spatial importation of SARS-CoV-2 infections such as from New York City to Connecticut, and may have over-estimated the magnitude of self-sustaining epidemic in locations receiving sustained SARS-Cov-2 importations. However, we also note that the Connecticut seroprevalence estimates predict an infection to observed case ratio that is substantially below those of the other CDC seroprevalence studies. The inferred contact patterns were assessed against external data from the BICS study that quantified human contact patterns during the pandemic (*9*) Validation results are reported in the supplementary materials, suggesting similarly strong reductions in human contact intensities as in the survey data. Disaggregated by age, the model reproduces highest contact intensities among 35-44 year old individuals, comparatively lower contact intensities from individuals aged 45+, and largest reductions in contact intensities from individuals aged 25-34. The survey data suggest that contact intensities from individuals aged 18-24 could be higher than reconstructed through the contact-and-infection model, but we also note large confidence intervals around the survey estimates.

Sensitivity analyses were conducted to assess central modelling assumptions on the infection fatality ratio, contact intensities among children and teens during periods of school closure, relative susceptibility of children and teens to SARS-CoV-2 infection, and are reported in the supplementary materials. Our findings on the age groups that drive SARS-CoV-2 transmission were found to be robust to these assumptions.

## Supplementary Materials

science.sciencemag.org/cgi/content/full/science.abe8372/DC1 Supplementary Text S1 to S6 Figs. S1 to S160 Tables S1 to S13 References (*51**–**119*) State-Level Situation Reports MDAR Reproducibility Checklist
